# Nanocytological Field Carcinogenesis Detection to Mitigate Overdiagnosis of Prostate Cancer: A Proof of Concept Study

**DOI:** 10.1371/journal.pone.0115999

**Published:** 2015-02-23

**Authors:** Hemant K. Roy, Charles B. Brendler, Hariharan Subramanian, Di Zhang, Charles Maneval, John Chandler, Leah Bowen, Karen L. Kaul, Brian T. Helfand, Chi-Hsiung Wang, Margo Quinn, Jacqueline Petkewicz, Michael Paterakos, Vadim Backman

**Affiliations:** 1 Department of Medicine, Boston University Medical Center, Boston, Massachusetts, United States of America; 2 Department of Surgery, NorthShore University HealthSystem, Evanston, Illinois, United States of America; 3 Biomedical Engineering Department, Northwestern University, Evanston, Illinois, United States of America; 4 Department of Pathology, NorthShore University HealthSystem, Evanston, Illinois, United States of America; University of Kentucky College of Medicine, UNITED STATES

## Abstract

**Purpose:**

To determine whether nano-architectural interrogation of prostate field carcinogenesis can be used to predict prognosis in patients with early stage (Gleason 6) prostate cancer (PCa), which is mostly indolent but frequently unnecessarily treated.

**Materials and Methods:**

We previously developed partial wave spectroscopic microscopy (PWS) that enables quantification of the nanoscale intracellular architecture (20–200nm length scale) with remarkable accuracy. We adapted this technique to assess prostate needle core biopsies in a case control study from men with Gleason 6 disease who either progressed (n = 20) or remained indolent (n = 18) over a ~3 year follow up. We measured the parameter disorder strength (Ld) characterizing the spatial heterogeneity of the nanoscale cellular structure and nuclear morphology from the microscopically normal mucosa ~150 histologically normal epithelial cells.

**Results:**

There was a profound increase in nano-architectural disorder between progressors and non-progressors. Indeed, the Ld from future progressors was dramatically increased when compared to future non-progressors (1±0.065 versus 1.30±0.0614, respectively p = 0.002). The area under the receiver operator characteristic curve (AUC) was 0.79, yielding a sensitivity of 88% and specificity of 72% for discriminating between progressors and non-progressors. This was not confounded by demographic factors (age, smoking status, race, obesity), thus supporting the robustness of the approach.

**Conclusions:**

We demonstrate, for the first time, that nano-architectural alterations occur in prostate cancer field carcinogenesis and can be exploited to predict prognosis of early stage PCa. This approach has promise in addressing the clinically vexing dilemma of management of Gleason 6 PCa and may provide a paradigm for dealing with the larger issue of cancer overdiagnosis.

## Introduction

Overdiagnosis is an emerging deterrent towards implementation of cancer population screening[1]. The detection of indolent/clinically insignificant disease frequently triggers a myriad of harms from unnecessary diagnostic/therapeutic interventions including costs, discomfort and complications[2]. Prostate cancer (PCa) epitomizes this conundrum in that it results in ~30,000 deaths in Americans, but ~60% of all men >80 years old incidentally harbor this malignancy[3]. The widespread use of serum prostate specific antigen (PSA) for PCa screening has accentuated detection of clinically-indolent cancers. Since the harms of treating early-stage (T1c/T2a), low-grade (Gleason 6) disease (the most common PCa presentation) generally outweigh the benefits, many clinical societies now recommend delaying surgery in favor of active surveillance, which involves close monitoring with serial prostate biopsies[4]. Unfortunately, given the vagaries of predicting behavior of an individual’s PCa (with the potential progression to fatal disease), only ~10% of eligible men actually undergo active surveillance[5]. Therefore, better prognostication tools are urgently needed to make this strategy more acceptable to both patients and their physicians[6].

One emerging approach for risk assessment is through exploiting field carcinogenesis. Field carcinogenesis (a.k.a. field cancerization, etiological field effect, field of injury etc.) is a well-established phenomenon in cancer biology that a focal neoplastic lesion develops in a permissive mutational environment from the interactions between genetic substrate and exogenous factors (diet, obesity, smoking, diabetes etc.) [7–9]. These changes may occur in the epithelial and/or stroma [10]. From a clinical perspective, field cancerization with the associated synchronous/metachronous lesions is widely used for management of neoplasia in the colon, lung, head and neck etc. While less well explored in prostate, there is compelling evidence of field carcinogenesis such as multi-focality and abnormalities in molecular markers (e.g. gene expression, methylation, microRNA, FISH, mitochondrial alterations) seen in the microscopically normal epithelium in patients who harbor this malignancy. [11–15]

Our group has developed a novel optics technology, partial wave spectroscopic microscopy (PWS), or nanocytology, which allows quantification of cellular structure at the nanometer length scales.[16] We have utilized PWS nanocytology for detection of the nano-architectural consequences of the diffuse genetic/epigenetic alterations that are the hallmark of field carcinogenesis. We have previously demonstrated that PWS interrogation of normal epithelium accurately predicted risk of colon[17] lung [18], esophagus[19], pancreas [20]and ovarian cancers[21]. To adapt this biophotonic breakthrough to PCa, we wanted to target the most vexing issue, management of early stage (Gleason 6 disease). Since this is typically diagnosed by needle biopsy, we hypothesized that nanocytological interrogation of the microscopically normal prostatic epithelium would predict prognosis in men with early stage (Gleason 6) disease undergoing active surveillance.

## Materials and Methods

### Patients

This study was approved by the Institutional Review Board at NorthShore University HealthSystem. Samples were obtained from the NorthShore University active surveillance trial initiated in November 2008. Informed written consent was obtained from the participants. The inclusion criteria for the cohort of patients included: 1) age ≥60; 2) clinical stage ≤T2a; 3) Gleason score (grade) <7; 4) ≤3/12 positive cores on initial biopsy; and 5) < 50% linear involvement of any single core. Patients underwent their first surveillance biopsy 6–12 months after enrollment. Progression was defined as any change in criteria 3, 4 or 5. Thirty-eight patients were randomly chosen from the database of patients adjudicated as progressors and non-progressors by the chief study urologist (CBB).

### Sample Procurement and Preparation

Transrectal biopsies were obtained under three dimensional (3-D) ultrasound guidance. Four of these cores were designated as research based and were fixed in an alcohol-based fixative and paraffin-embedded. Four micrometer sections were cut onto charged glass slides and deparaffinized for PWS. Hematoxylin and eosin (H&E) sections were reviewed by the study pathologist (MP) to direct the PWS analysis towards non-malignant tissue.

### Overview of PWS

Briefly, as previously described, PWS marker disorder strength (Ld) is proportional to the mean and standard deviation of the spatial variations of the macromolecular density of the fundamental cellular building blocks (proteins, nucleic acids, lipids). Thus, Ld, colloquially, can be described as measuring the “clumpiness” of nanoscale structure. We have demonstrated that PWS is sensitive to structures from 20–200 nm through the spectral analysis of the interference spectra of light reflected from intracellular refractive index variations within microscopic spatio-temporal coherence volume, as opposed to typical light microscopy whose resolution is restricted 200–500 nm, the diffraction limit of light. Thus, histology is insensitive to many structures known to be involved in early carcinogenesis (macromolecular complexes, ribosomes, mitochondria, high-order chromatin etc.) that may be PWS detectable. Previously, we have shown that PWS was able to predict risk of colon, lung, esophagus, pancreas and ovarian cancers[22].

### PWS Data Acquisition and Analysis

PWS analysis was performed as previously described. Regions of interest were identified (histologically normal glands) by the pathologist and calculated over ~30 regions per patient slide examined (~50K-75K pixels or ~150 cells). The operator did not have clinical information for any of the 38 samples. However, the clinical information was available to technological investigators in the first 18 (“unblinded”) but not the last 20 (“blinded”) samples was performed in the first 18 patients but not the last 20.

### Statistical Methods

All statistical analyses were performed using SAS 9.3 (SAS Inc., Cary, NC). Patient demographics were compared between progressors and non-progressors using t-test (for continuous variables) and Chi-square test. A standard two tailed t-test (assuming unequal variances) and analysis of covariance (ANCOVA) were performed to compare the Ld between progressors and non-progressors with a p value ≤0.05 considered statistically significant. The effect size was calculated using Cohen’s *d*: $Effect\;size\;=\;\mu_{1}\mu_{2}/\sqrt{\sigma_{1}^{2}+\sigma_{2}^{2}}$ where *μ*_1_ and *μ*_2_ are the means for progressors and non-progressors and *σ*_1_ and *σ*_2_ are the corresponding standard deviations, respectively. The study statistician (CHW) also performed receiver operator characteristic curve (ROC) to assess the sensitivity and specificity of PWS in differentiating progressors and non-progressors for PCa in active surveillance.

## Results

### Cohort

**Fig 1:**
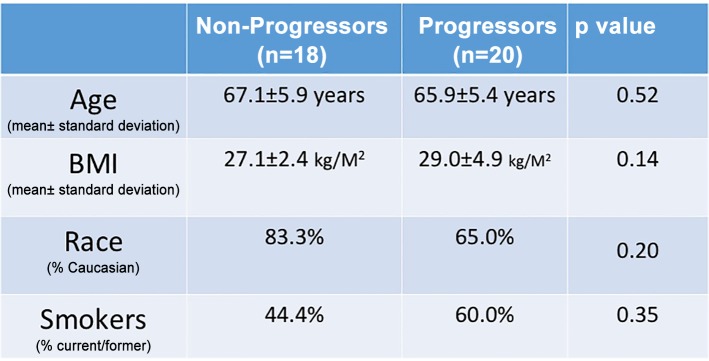
Relevant demographic characteristics at time of first surveillance biopsy for patients who were later determined to be progressors versus non-progressors.

We enrolled two sets of patients, an initial cohort of 18 patients (8 non-progressors and 10 progressors) where the PWS operator was blinded to clinical status but, followed by a distinct group of 20 patients (10 progressor and 10 non-progressors) in which our PWS analysis was performed by investigators blinded to group. The demographic factors are listed in Fig. 1. Median length of follow up was 34.2 months, mean age 66.5 ±5.6 years, and mean BMI 28.1±4.0. There were no significant differences in baseline patient characteristics between progressors and non-progressors.

### PWS Analysis of Prostate Tissue Sections

**Fig 2:**
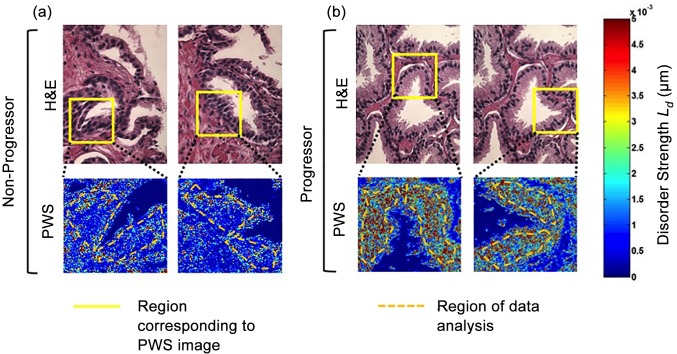
Representative images of prostate sections with bright field microscopy and Ld pseudocolor image maps. The bright field images from non-progressors (a) and progressors (b) are indistinguishable on the initial surveillance biopsies. However, the PWS images in the progressors show markedly higher Ld (e.g. red areas) when compared to the non-progressors, indicating profound nano-architectural abnormalities in the pathologically normal prostate epithelium. Median length of follow up was 34.2 months.

PWS was performed in histologically normal glands identified by our pathologist. As can be noted from bright field imaging, we primarily targeted epithelium adjacent to glands for the defined region of interest. While there was no apparent difference on the bright field, there was a profound increase in Ld (more red in pseudocolor map) in progressors versus non-progressors (Fig. 2).

### Mean per patient Ld in Progressors versus NonProgressors

**Fig 3:**
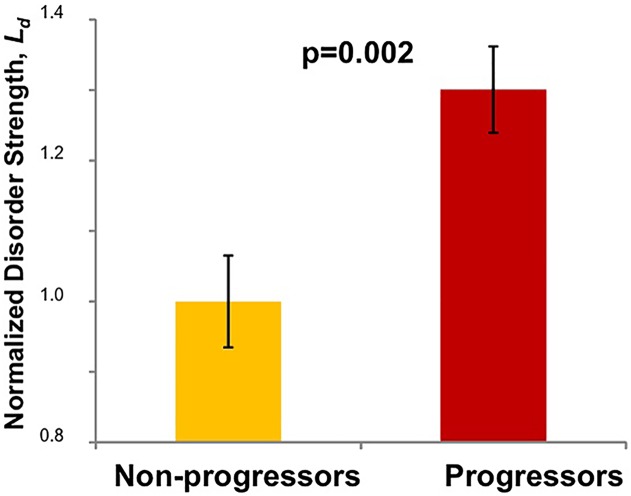
(a) Mean disorder strength (Ld) in non-progressors (n = 18) and progressors (n = 20) show a statistically significant difference between the two patient groups (p = 0.002) with an effect size of diagnosis = 110%. The median follow up time was 34.2 months.

**Fig 4:**
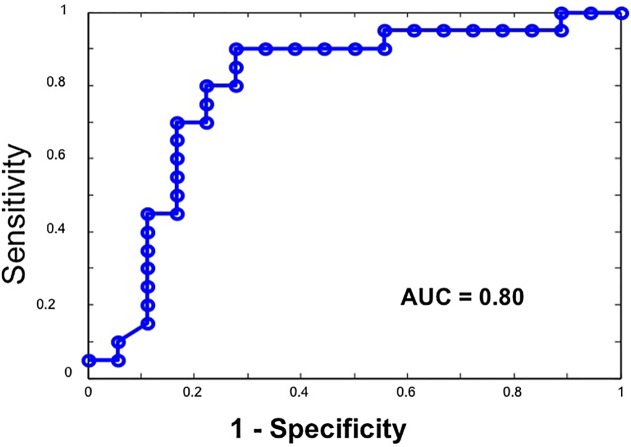
The ROC curve obtained from the composite dataset (n = 38) demonstrates an area under curve (AUC) of 0.80. This translated into a sensitivity = 88% and specificity = 72% for discriminating between progressors and non-progressors.

We calculated the mean Ld from the region of interest per patient. Elevated Ld has been a hallmark of neoplastic transformation in a number of organs[20]. The Cohen d effect size of mean cellular Ld in this case-control study was 110% (Fig. 3) with a significant difference between progressors and non-progressors (p = 0.002). We analyzed half the dataset in a blinded fashion and noted that the AUC was equivalent to unblinded samples (0.81 versus 0.75, respectively), supporting the robustness of methodology. The combined dataset yielded a sensitivity of 88% and specificity of 72%, (Fig. 4) while the blinded dataset alone (n = 20) ad a sensitivity of 100% and a specificity of 70%. These strong diagnostics represent the minimal performance characteristics, with future refinements (e.g. assaying separately cytoplasmic versus nuclear Ld and epithelial versus stromal Ld) promising to improve discriminatory ability.

### Potential Confounders

Since clinical factors (e.g. age, race[23,24], BMI[25], and smoking[26]) may modulate PCa aggressiveness, we performed analyses of covariance (ANCOVA) comparing Ld between progressors and non-progressors while controlling for these potential confounders. Somewhat surprisingly (based on previous literature) [27], serum prostate specific antigen was noted to be more elevated in progressors than nonprogressors, although statistical analysis showed that this did not impact upon Ld’s diagnostic ability.

## Discussion

The clinical need to differentiate aggressive from indolent PCa is clear since it is estimated that 233,000 American men are diagnosed annually with PCa, but only 29,480 are projected to die (still the second leading cause of male cancer deaths)[28]. While PSA screening has been widely practiced, the harms associated with the diagnosis and treatment of clinically indolent disease have led the US Preventive Services Task force to recently recommend against PSA screening[29].

The issue of overdiagnosis now presents a major impediment to cancer screening in other cancers as well, including mammography for breast cancer and low-dose computerized tomography (LDCT) scanning for lung cancer[1]. Indeed, while LDCT resulted in a 20% reduction in lung cancer deaths in a high risk population, >95% of positives were false positive[30]. Importantly, nanocytological field carcinogenesis detection has been demonstrated to be useful in screening for a variety of cancers that manifest field carcinogenesis (colon, lung, esophagus, pancreas, ovarian etc)[19–22,31]. One of the major novel aspects of this study is that it is the first to not simply assess presence/absence of tumor (screening), but rather prognosis. This has been fostered by our ability to overcome technical challenges and actually perform PWS nanocytology on intact tissue sections (the previously reported studies were all from cytological brushings). Thus, PWS nanocytology represents a platform technology that is applicable to screening/prognostication of a large number of malignancies.

Our approach of exploiting field carcinogenesis is particularly apropos for PCa given its multifocal nature. Indeed, the Gleason score is calculated by assessing the grade of the most common and next most common (or most aggressive) clone. While assessing individual tumor clones (e.g. 3-gene immunohistochemical signature) may correlate with the natural history of some PCa[32], given the clonal heterogeneity of most PCa and the semi-quantitative nature of these tests, they may have limited clinical utility. Intuitively, assaying field carcinogenesis within prostate specimens with or without PCa is potentially more attractive than focusing on genetic signatures obtained from specimens containing only PCa, as currently offered by tests such as Oncotype Dx, Prolaris, Decipher etc. [33]. There have been numerous other field effect markers (methylation, microRNA, gene expression) with putative prognostic ability, albeit performance to date has been modest[13,33,34]. Assessing nano-architecture is particularly powerful since it reflects the final common denominator beyond converging disparate molecular pathways. While other modalities (transmission electron microscopy[35] or karyometry[36] have noted abnormalities, their utilization as clinical tools are not feasible. The ability to distill PWS information into single biomarker (Ld) has considerable appeal from both a practicality and robustness perspective. Moreover, PWS quantifies the nano-architecture of specific compartments as well as throughout the cell.

Our previous work on nanocytological field carcinogenesis has been to assess presence of neoplasia; thus, this report represents a major new application for this approach, i.e. prognostication. Intuitively, it does seem logical to believe that field carcinogenesis profiling may be more promising than focusing on the tumor alone because of the multi-clonality that is the hallmark of prostate cancer. Indeed, the Gleason score is based on the grade of the most common and then either the next most common or most aggressive clone. Thus, it would be unclear which clone to analyze or how to synthesize gene expression data from a variety of clones. Our work parallels some of the data with liver cancer (another frequently multifocal disease) that profiling the uninvolved hepatocytes predicted recurrence in the liver after resection. [37]

Biologically, increased cellular nano-architectural disorder is intimately related to molecular events. From a physical perspective, an increased Ld implies “clumpiness” at the 20–200 nm length scales. These length scales encompass structures ranging from macromolecular complexes to small organelles. In the nucleus, disorder strength (Ld) is a measure of high order chromatin organization, which impacts upon multiple processes controlling gene expression.,, In keeping this this, we have demonstrated that nuclear Ld correlates with transcriptional activity[38]. In field carcinogenesis it is well established that gene expression is altered[37,39]. Furthermore, various gene alterations have been shown to be altered in prostate field carcinogenesis. However, given the genetic heterogeneity in tumors, finding the precise gene array is difficult. Since it appears that nano-architecture may be a “final common denominator” to the myriad of genetic/epigenetic alterations in early carcinogenesis, it should be particularly powerful. [20] In the cytoplasm, the determinants are more varied but an overarching theme is cytoskeletal organization[40]. Numerous cytoskeletal proteins (e.g. smooth muscle gamma actin) have been shown to be dysregulated in prostatic epithelium, further supporting the relevance to PCa[41].

There are many strengths of this study including its innovative nature and the well-characterized, prospectively followed cohort of men undergoing active surveillance. The clinical novelty includes use of field carcinogenesis to predict natural history of early stage PCa. From a biological perspective, this provides important insights into nano-architectural abnormalities in early carcinogenesis. Technologically, this study not only used PWS to assess prognosis, rather than just the presence of tumor, but also overcame the challenge of performing nanocytology on whole tissue sections.

Weaknesses that need to be acknowledged include modest cohort size and follow-up. On the other hand, this study represents the gold standard with the samples collected in a PRoBe-like fashion (samples prospectively collected and last half of dataset blinded; given that even in the unblinded dataset the investigator responsible for data acquisition was unaware of the clinical status, there is no possibility of bias). Another potential weakness of this study was the lack of clinically unequivocal endpoints, e.g. metastasis or death from PCa. Both of these endpoints, however, would require at least 10 years of follow up and would therefore be impractical. Furthermore, our definition of progression (increased Gleason score or increased volume of cancer) is well validated and widely used in clinical practice.

In conclusion, we demonstrate there are profound nano-architectural alterations in prostate cancer field carcinogenesis. Assessment of this through PWS nanocytology may represent a powerful biomarker to predict progression for men with early stage PCa. This approach may make PCa population screening more viable by mitigating the harms associated with identification of indolent disease. Moreover, this proof of principle study suggests that PWS nanocytology may potentially have a role in the clinical armamentarium against the screening overdiagnosis conundrum for a number of malignancies.

## References

[1] Esserman LJ, Thompson IM Jr., Reid B (2013) Overdiagnosis and overtreatment in cancer: an opportunity for improvement. JAMA 310: 797–798. 23896967 10.1001/jama.2013.108415

[2] Walsh PC, DeWeese TL, Eisenberger MA (2007) Clinical practice. Localized prostate cancer. N Engl J Med 357: 2696–2705. 18160689 10.1056/NEJMcp0706784

[3] Zlotta AR, Egawa S, Pushkar D, Govorov A, Kimura T, et al (2013) Prevalence of prostate cancer on autopsy: cross-sectional study on unscreened Caucasian and Asian men. J Natl Cancer Inst 105: 1050–1058. 10.1093/jnci/djt151 23847245

[4] Carter HB (2013) American Urological Association (AUA) guideline on prostate cancer detection: process and rationale. BJU Int 112: 543–547. 10.1111/bju.12318 23924423

[5] Ganz PA, Barry JM, Burke W, Col NF, Corso PS, et al (2012) National Institutes of Health State-of-the-Science Conference: role of active surveillance in the management of men with localized prostate cancer. Ann Intern Med 156: 591–595. 10.7326/0003-4819-156-8-201204170-00401 22351514 PMC4774889

[6] Latini DM, Hart SL, Knight SJ, Cowan JE, Ross PL, et al (2007) The relationship between anxiety and time to treatment for patients with prostate cancer on surveillance. J Urol 178: 826–831; discussion 831–822. 17632144 10.1016/j.juro.2007.05.039

[7] Backman V, Roy HK (2011) Light-scattering technologies for field carcinogenesis detection: a modality for endoscopic prescreening. Gastroenterology 140: 35–41. 10.1053/j.gastro.2010.11.023 21078318 PMC3319699

[8] Steiling K RJ, Broady JS, Spira A (2008) the field of tissue injury in the lung and airway. Cancer Prev Res 1: 396–403. 10.1158/1940-6207.CAPR-08-0174 19138985 PMC2705781

[9] Lochhead P, Chan AT, Nishihara R, Fuchs CS, Beck AH, et al. (2014) Etiologic field effect: reappraisal of the field effect concept in cancer predisposition and progression. Mod Pathol. 10.1038/modpathol.2014.81 PMC4265316 24925058

[10] Dotto GP (2014) Multifocal epithelial tumors and field cancerization: stroma as a primary determinant. J Clin Invest 124: 1446–1453. 10.1172/JCI72589 24691479 PMC3973113

[11] Kosari F, Cheville JC, Ida CM, Karnes RJ, Leontovich AA, et al (2012) Shared gene expression alterations in prostate cancer and histologically benign prostate from patients with prostate cancer. Am J Pathol 181: 34–42. 10.1016/j.ajpath.2012.03.043 22640805 PMC3388167

[12] Luo JH, Ding Y, Chen R, Michalopoulos G, Nelson J, et al (2013) Genome-wide methylation analysis of prostate tissues reveals global methylation patterns of prostate cancer. Am J Pathol 182: 2028–2036. 10.1016/j.ajpath.2013.02.040 23583283 PMC3668028

[13] Nonn L, Ananthanarayanan V, Gann PH (2009) Evidence for field cancerization of the prostate. Prostate 69: 1470–1479. 10.1002/pros.20983 19462462 PMC3690597

[14] Parr RL, Mills J, Harbottle A, Creed JM, Crewdson G, et al (2013) Mitochondria, prostate cancer, and biopsy sampling error. Discov Med 15: 213–220. 23636138

[15] Zhang Y, Perez T, Blondin B, Du J, Liu P, et al (2014) Identification of FISH biomarkers to detect chromosome abnormalities associated with prostate adenocarcinoma in tumour and field effect environment. BMC Cancer 14: 129 10.1186/1471-2407-14-129 24568597 PMC4016502

[16] Subramanian H, Pradhan P, Liu Y, Capoglu IR, Li X, et al (2008) Optical methodology for detecting histologically unapparent nanoscale consequences of genetic alterations in biological cells. Proc Natl Acad Sci U S A 105: 20118–20123. 10.1073/pnas.0804723105 19073935 PMC2629261

[17] Damania D, Roy HK, Subramanian H, Weinberg DS, Rex DK, et al (2012) Nanocytology of rectal colonocytes to assess risk of colon cancer based on field cancerization. Cancer Res 72: 2720–2727. 10.1158/0008-5472.CAN-11-3807 22491589 PMC3557939

[18] Roy HK, Subramanian H, Damania D, Hensing TA, Rom WN, et al (2010) Optical detection of buccal epithelial nanoarchitectural alterations in patients harboring lung cancer: implications for screening. Cancer Res 70: 7748–7754. 10.1158/0008-5472.CAN-10-1686 20924114 PMC3703950

[19] Konda VJ, Cherkezyan L, Subramanian H, Wroblewski K, Damania D, et al (2013) Nanoscale markers of esophageal field carcinogenesis: potential implications for esophageal cancer screening. Endoscopy 45: 983–988. 10.1055/s-0033-1344617 24019132 PMC4195538

[20] Subramanian H, Roy HK, Pradhan P, Goldberg MJ, Muldoon J, et al (2009) Nanoscale cellular changes in field carcinogenesis detected by partial wave spectroscopy. Cancer Res 69: 5357–5363. 10.1158/0008-5472.CAN-08-3895 19549915 PMC2802178

[21] Damania D, Roy HK, Kunte D, Hurteau JA, Subramanian H, et al (2013) Insights into the field carcinogenesis of ovarian cancer based on the nanocytology of endocervical and endometrial epithelial cells. Int J Cancer 133: 1143–1152. 10.1002/ijc.28122 23436651 PMC3695064

[22] Backman V, Roy HK (2013) Advances in biophotonics detection of field carcinogenesis for colon cancer risk stratification. J Cancer 4: 251–261. 10.7150/jca.5838 23459690 PMC3584838

[23] Jalloh M, Myers F, Cowan JE, Carroll PR, Cooperberg MR (2014) Racial Variation in Prostate Cancer Upgrading and Upstaging Among Men with Low-risk Clinical Characteristics. Eur Urol. 10.1016/j.eururo.2014.03.026 24746973

[24] Sundi D, Kryvenko ON, Carter HB, Ross AE, Epstein JI, et al (2014) Pathological examination of radical prostatectomy specimens in men with very low risk disease at biopsy reveals distinct zonal distribution of cancer in black American men. J Urol 191: 60–67. 10.1016/j.juro.2013.06.021 23770146 PMC4042393

[25] Gong Z, Agalliu I, Lin DW, Stanford JL, Kristal AR (2007) Obesity is associated with increased risks of prostate cancer metastasis and death after initial cancer diagnosis in middle-aged men. Cancer 109: 1192–1202. 17311344 10.1002/cncr.22534

[26] Kenfield SA, Stampfer MJ, Chan JM, Giovannucci E Smoking and prostate cancer survival and recurrence. Jama 305: 2548–2555. 10.1001/jama.2011.879 21693743 PMC3562349

[27] Vickers AJ, Thompson IM, Klein E, Carroll PR, Scardino PT (2014) A commentary on PSA velocity and doubling time for clinical decisions in prostate cancer. Urology 83: 592–596. 10.1016/j.urology.2013.09.075 24581521

[28] Siegel R, Ma J, Zou Z, Jemal A (2014) Cancer statistics, 2014. CA Cancer J Clin 64: 9–29. 10.3322/caac.21208 24399786

[29] Moyer VA, Force USPST (2012) Screening for prostate cancer: U.S. Preventive Services Task Force recommendation statement. Ann Intern Med 157: 120–134. 10.7326/0003-4819-157-2-201207170-00459 22801674

[30] Aberle DR, DeMello S, Berg CD, Black WC, Brewer B, et al (2013) Results of the two incidence screenings in the National Lung Screening Trial. N Engl J Med 369: 920–931. 10.1056/NEJMoa1208962 24004119 PMC4307922

[31] Roy HK, Hensing T, Backman V (2011) Nanocytology for field carcinogenesis detection: novel paradigm for lung cancer risk stratification. Future Oncol 7: 1–3. 10.2217/fon.10.176 21174531 PMC4123752

[32] Irshad S, Bansal M, Castillo-Martin M, Zheng T, Aytes A, et al (2013) A molecular signature predictive of indolent prostate cancer. Sci Transl Med 5: 202ra122 10.1126/scitranslmed.3006408 24027026 PMC3943244

[33] Sartori DA, Chan DW (2014) Biomarkers in prostate cancer: what's new? Curr Opin Oncol 26: 259–264. 10.1097/CCO.0000000000000065 24626128 PMC4110681

[34] Trujillo KA, Jones AC, Griffith JK, Bisoffi M (2012) Markers of field cancerization: proposed clinical applications in prostate biopsies. Prostate Cancer 2012: 302894 10.1155/2012/302894 22666601 PMC3361299

[35] Montironi R, Filho AL, Santinelli A, Mazzucchelli R, Pomante R, et al (2000) Nuclear changes in the normal-looking columnar epithelium adjacent to and distant from prostatic intraepithelial neoplasia and prostate cancer. Morphometric analysis in whole-mount sections. Virchows Arch 437: 625–634. 11193474 10.1007/s004280000290

[36] Campos-Fernandes JL, Bastien L, Nicolaiew N, Robert G, Terry S, et al (2009) Prostate cancer detection rate in patients with repeated extended 21-sample needle biopsy. Eur Urol 55: 600–606. 10.1016/j.eururo.2008.06.043 18597923

[37] Hoshida Y, Villanueva A, Kobayashi M, Peix J, Chiang DY, et al (2008) Gene expression in fixed tissues and outcome in hepatocellular carcinoma. N Engl J Med 359: 1995–2004. 10.1056/NEJMoa0804525 18923165 PMC2963075

[38] Tiwari AK (2013) Partial Wave Spectroscopic Microscopy: A Novel Tool to Assess Perturbation in teh Cellular Transcriptional Activity During Colon Carcinogenesis. Gastroenterology 144: 1.

[39] Chen LC, Hao CY, Chiu YS, Wong P, Melnick JS, et al (2004) Alteration of gene expression in normal-appearing colon mucosa of APC(min) mice and human cancer patients. Cancer Res 64: 3694–3700. 15150130 10.1158/0008-5472.CAN-03-3264

[40] Damania D, Subramanian H, Tiwari AK, Stypula Y, Kunte D, et al (2010) Role of cytoskeleton in controlling the disorder strength of cellular nanoscale architecture. Biophys J 99: 989–996. 10.1016/j.bpj.2010.05.023 20682278 PMC2913198

[41] Fillmore RA, Kojima C, Johnson C, Kolcun G, Dangott LJ, et al (2014) New concepts concerning prostate cancer screening. Exp Biol Med (Maywood) 239: 793–804. 24928864 10.1177/1535370214539091

